# Minimally invasive tubular removal of spinal schwannoma and neurofibroma - a case series of 49 patients and review of the literature

**DOI:** 10.1007/s10143-024-02656-x

**Published:** 2024-08-10

**Authors:** Katerina Argiti, Ralf Watzlawick, Marc Hohenhaus, Ioannis Vasilikos, Florian Volz, Roland Roelz, Christoph Scholz, Ulrich Hubbe, Jürgen Beck, Matthias Neef, Jan-Helge Klingler

**Affiliations:** 1 Department of Neurosurgery, University Medical Center Freiburg, Breisacher Straße 64, D-79106 Freiburg, Germany; 2 Department of Neurosurgery, University Hospital Knappschaftskrankenhaus Bochum, Bochum, Germany

**Keywords:** Schwannoma, Neurofibroma, Minimally invasive, Spine surgery, Nerve sheath tumor

## Abstract

To evaluate the efficacy and safety of minimally invasive tubular removal of spinal schwannoma and neurofibroma. In this single-centre study, we retrospectively analysed 49 consecutive patients who underwent minimally invasive removal of a total of 51 benign spinal nerve sheath tumors using a non-expandable (*n* = 18) or expandable tubular retractor (*n* = 33) retractor system between June 2007 and December 2019. The extent of resection, surgical complications, neurological outcome, operative time, and estimated blood loss were recorded. Histopathology revealed 41 schwannomas and 10 neurofibromas. After a mean follow-up of 30.8 months, postoperative MRI showed gross total resection in 93.7%, and subtotal resection in 6.3% of the tumors. Three patients were lost to follow up. Of the subtotal resections, one was a schwannoma (2.4% subtotal resections in schwannomas) and two were neurofibromas (20.0% subtotal resections in neurofibromas). Intraspinal and paraspinal tumor localizations were equally accessible by minimally invasive tubular surgery. Conversion to open surgery was not required in any case. The mean operative time was 167 ± 68 min, and estimated blood loss was 138 ± 145 ml. We observed no major surgical complications. Spinal schwannoma and neurofibroma can be removed effectively and safely using a minimally invasive tubular approach, with satisfying extent of tumor resection comparable to the conventional open surgical technique and no increased risk for neurological deterioration.

**Supplementary Information:**

The online version contains supplementary material available at 10.1007/s10143-024-02656-x.

## Introduction

Spinal tumors occur with an incidence of 1–2/100.000 [1]. Benign spinal nerve sheath tumors (BNST) such as schwannoma and neurofibroma are the most frequent spinal neoplasms accounting for 30% f all spinal tumors [1]. 75% of BNST are intradural, 10–15% mixed intra- and extradural and 10–15% completely extradural [2, 3]. The so-called dumbbell tumor shows intra- and extraspinal growth through the neuroforamen.

Symptoms of BNST caused by compression of nerve roots or the spinal cord comprise local or radiating pain, neurological deficits, and myelopathy of varying severity [1]. In addition to clinical examination, contrast-enhanced magnetic resonance image (MRI) is the gold standard imaging modality.

Conventionally, therapy consists of open surgery with the goal of a gross total resection (GTR) of the tumor. Therefore, preparation of the paravertebral musculature and hemilaminectomy are usually necessary. Partial or complete facetectomy is often unavoidable. As these lesions are benign, patients undergoing complete surgical excision have a favourable outcome.

In spine surgery in general, minimally invasive surgery (MIS) techniques have become increasingly popular within the past three decades [4]. Evidence-based advantages of MIS over conventional open surgery comprise a reduction in blood loss, pain medication during and after surgery, a shorter inpatient stay, faster return to work and cost effectiveness for the health care system [5–10]. In view of these benefits, we introduced MIS procedures for the treatment of BNST in 2007 [11].

The objective of this study is to evaluate the efficacy and safety of MIS tubular removal of spinal and paraspinal schwannoma and neurofibroma.

## Materials and methods

### Data collection

This single-centre retrospective data analysis included 51 minimally invasive removals of spinal and paraspinal BNST in 49 patients. Surgeries were performed between June 2007 and December 2019. Inclusion criteria were MIS employing a nonexpandable or expandable tubular retractor and the histological confirmation of BNST (schwannoma or neurofibroma). This study was approved by the local ethics committee (98/13) and is registered in the German Clinical Trials Register (DRKS00004842).

**Fig. 1:**
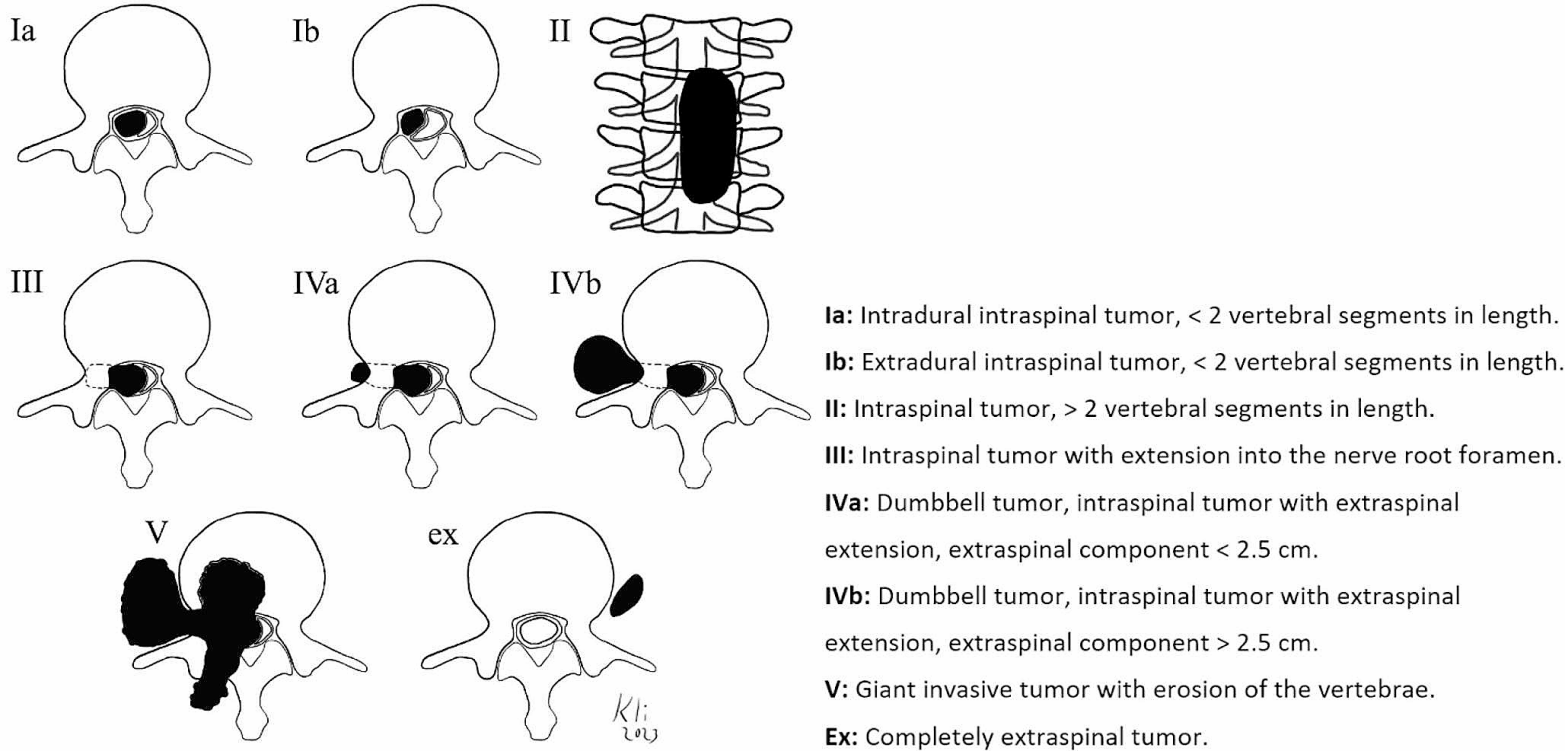
Modified Sridhar classification, based on Sridhar et al. 2001 [12]. (Figures: J.-H. Klingler)

We collected details on tumor size, location (Sridhar classification, Fig. 1, [12]), extent of the tumors from preoperative imaging and details on the surgical technique, estimated blood loss, operative time, pre- and postoperative neurological status as well as surgical complications from medical records [12]. In addition, pain relief was determined using the numeric pain rating scale (NRS). Postoperative follow-up MRI was used to assess the extent of resection.

### Surgical technique

**Fig. 2:**
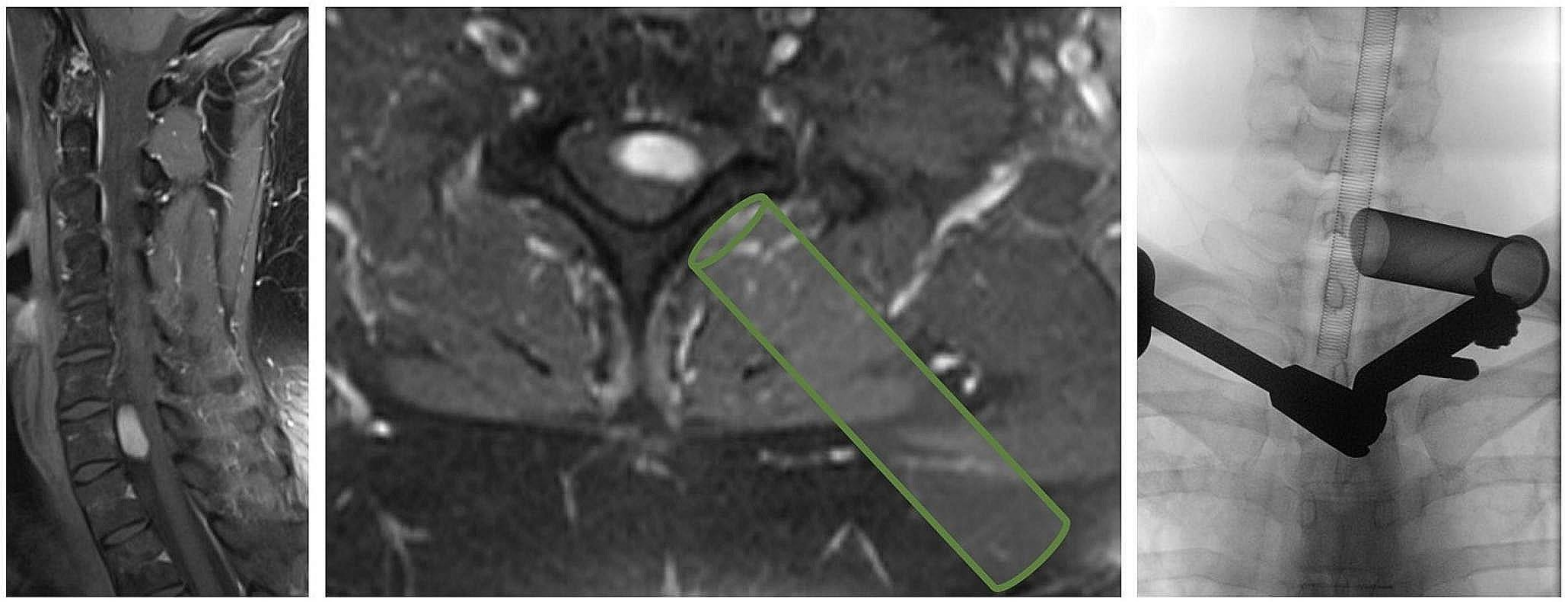
Exemplary case of an intraspinal intradural BNST anterior to the spinal cord at the level of C7 on contrast enhanced T1 weighed MRI (left). Axial MRI shows superimposed the minimally invasive tubular approach in green (middle). Intraoperative anterior-posterior X-ray of the tubular retractor placement (right)

All patients underwent minimally invasive unilateral spinal tumor resection. Therefore, each patient was placed in prone position under general anesthesia. Intraoperative neurophysiological monitoring was performed individually when needed. The most used technique was direct nerve stimulation. If needed, sensory evoked potentials or motor evoked potentials were used. The level of interest was determined using fluoroscopy. Through a small skin incision, a minimally invasive surgical corridor to the spine was established with trans muscular dilatators, and a definitive nonexpandable or expandable surgical tube (18–20 mm diameter METRx_TM_ tubular retractor system or 22 mm diameter MAST QUADRANT™ retractor system, both Medtronic, Minneapolis, USA) was placed under fluoroscopic control (Fig. 2). In the case of an intraspinal intradural tumor, posterior exposure of the spinal canal was performed with gentle access to the dura and the exiting nerve root. This was achieved by minimized removal of the dorsal bony structures tailored to the extent of the individual tumor, usually by partial hemilaminectomy, while sparing the facet joint as much as possible. The bone is removed by using a high speed drill. For further preparation of the tissue in depth, bayonet style instruments are required. Sharp longitudinal durotomy was performed either by scalpel or sharp scissors. Two tenting sutures were placed on each side of the durotomy and tension was applied. Here fore, double tubular technique can be used to achieve a further expansion of the exposure of the intradural structures through the same durotomy [13]. Direct nerve stimulation was used to identify and spare motory nerves. Resection of the tumor was carried out under microscopic view. For tumor removal, bipolar, scissors, sharp hook and tumor forceps are utilized. After tumor removal, hemostasis was obtained through bipolar coagulation and hemostatic agents. Dural closure was performed using nonabsorbable, braided 5 − 0 sutures supported by fibrin glue and gelfoam or Tachosil^®^ (Takeda, Berlin, Germany). Watertightness was verified with a Valsalva maneuver. While slowly removing the tubular retractor under meticulous hemostasis of paraspinal muscles, the incision was closed with subcutaneous suture and skin adhesive without insertion of a subfascial drain. For further visualization of the surgical technique, an intraoperative video of an exemplary case was published along with this work.

For extraforaminal or paraspinal localized tumors, the tubular retractor system was guided as close as possible to the tumor under fluoroscopic control after trans muscular dilatation. The BNST was then excised under microscopic magnification while sparing the passing nerve fibers.

In MIS precise localization of the tumor via fluoroscopy is crucial, since the operating field is narrow and there are no exposed structures for anatomical orientation in situ. Especially in extraspinal tumors, measuring the distance to bony structures on CT/MRI scans before the surgery can aid a correct localization. Handling of bayonet style instruments requires expertise of the surgeon and should be practiced in less challenging procedures before applied on intradural tumor resection. Depending on the depth of the target structure, there are retractors in different lengths. Also, extra-long bayonet instruments can be used. It should be taken to consideration, that the handling of the instruments and the preparation of the tissue gets more difficult the deeper the target structure lays. Therefore, obesity can be challenging for a minimally invasive approach.

Apart from electrophysiological monitoring there are other technical appliances that can be used in MIS of BNST. In selected cases ultrasound can be useful to detect the optimal position for durotomy. However, depending on the available equipment, some ultrasound probes are too big to be fitted through a MIS tubular retractor. McGrath et al. described the application of 3-dimensional-navigated localization for MIS on intradural extramedullary spinal tumors as safe and effective [14]. We do not use 3-dimensional-navigated localization as a standard for spinal tumors without need for spinal instrumentation since 2-dimensional fluoroscopy is sufficient to localize each vertebra and exposes the patient to markedly less radiation. A 3-dimensional-navigated may prolong the surgery and therefore anesthesia duration with the resulting risks. For less experienced surgeons we see an advantage in navigated surgery to aid the assessment of the extend of bone resection and the preservation of the facet joint. In BNST we rarely use intraoperative dye since the tumors usually show clear borders. For identification of the motor nerve fibers, we use direct nerve stimulation.

### Statistical analysis

Descriptive statistics were calculated using Prism 6 for Mac (GraphPad Software Inc., La Jolla, USA). Normally distributed patient characteristics were presented as means with standard deviation (SD).

## Results

Forty-nine patients with a total of 51 BNST underwent MIS tumor removal between 2006 and 2019 (Table 1). During the same period, 54 BNST were resected via open approach. Two patients underwent two separate surgeries. In one of these patients a Th9 neurofibroma and a lumbar schwannoma were resected on the same day. The other patient underwent removal of L5 schwannoma first and of L1/2 schwannoma one year later. Of all patients, 22 were male (44.9%) and 27 female (55.1%). The median age was 51 years (SD ± 16.6, range 14–80 years). Body mass index varied from 19 to 40 kg/m^2^ (Table 1).

Patients presented with local or radiating pain (76.5%), sensory deficits (53.0%), motor deficits (29.5%) and/or other symptoms (6.0%). In 10% the diagnosis of the tumor was incidental. Mean symptom duration was 18.7 months (SD ± 31.0, range 0-180 months).

**Fig. 3:**
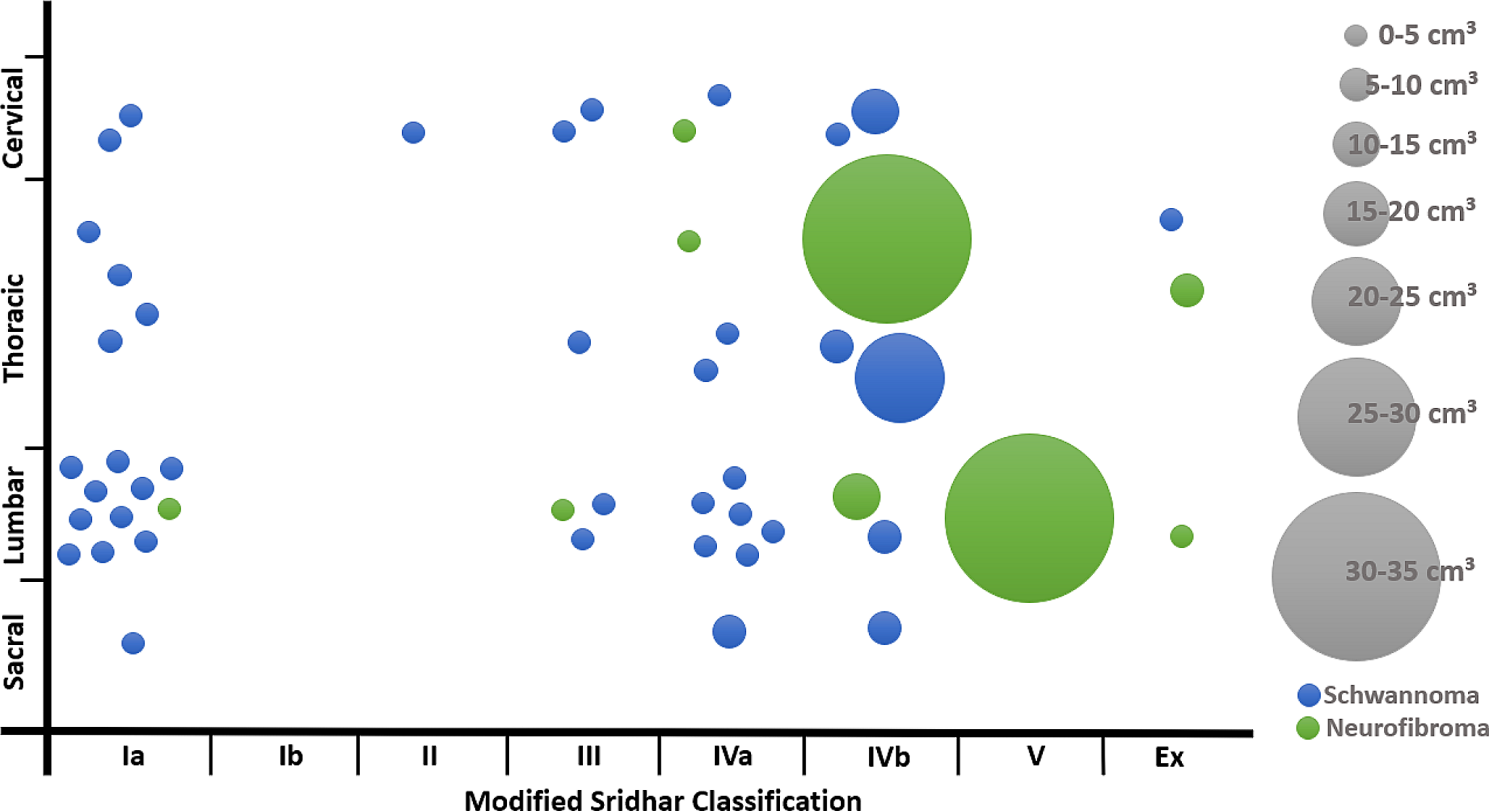
Tumor characteristics. X scale: Modified Sridhar classification, extended with category “ex” for extraspinal lesions. Y scale shows location of the tumor with cervical, thoracic, lumbar, and sacral. Each blue dot represents one schwannoma, each green dot a neurofibroma. The size of the dots represents the size of the lesion according to the categories shown on the right. Most lesions were small Ia lumbar schwannomas. However, our study also includes class IV and V tumors and giant tumors

56.9% of tumors were located completely intraspinal, 11.8% were extraspinal and 31.4% were dumbbell tumors (Supplement Table A, Fig. 2). 54.9% were intraoperatively classified as intradural, 35.3% as extradural and 9.8% as mixed. The cases included 13.7% cervical, 3.9% each cervicothoracic and lumbosacral, 15.7% thoracic, 11.8% thoracolumbar, 45.1% lumbar and 5.9% sacral tumor locations (Fig. 3). The mean volume was 4.2 cm³ (SD ± 6.9, range 0.1–34.6 cm³).

Operative time was 167 min (SD ± 68, range 70–364 min). Estimated blood loss was 138 ml (SD ± 145.5, range 50–800 ml). Intraoperative neurophysiological monitoring was performed in 68.6% of the cases (Supplement Table A). The size of the skin incisions was 3.1 cm (SD ± 0.8, range 1.5–4.5 cm). 35.3% of the surgeries were performed using the non expandable tubular and 64.7% using an expandable retractor system. No patient needed fusion surgery during the initial surgery or during the follow-up period of 30.8 months (SD ± 30.3, range 0-103 months). All spinal and paraspinal tumor surgeries were minimally invasive and could be successfully addressed surgically without converting to an open technique. Facetectomy was not required in any of the cases. Patients were successfully mobilized on the first postoperative day. In postoperative pain management none of the patients required patient-controlled analgesia. NRS decreased from 2.5 ± 2.5 preoperatively to 1.5 ± 1.7 at discharge. The postoperative length of hospital stay was 5.3 days (SD ± 2.0, range 2–12 days).

Histopathology revealed 41 schwannomas and 10 neurofibromas (Supplement Table B). 5.9% of the patients were lost to follow up. Out of the patients who received follow up postoperative MRI, 6.3% showed subtotal resection (STR), 93.7% showed GTR. Of the STR, one was a schwannoma (2.4% STR in schwannomas) and two were neurofibromas (20% STR in neurofibromas). In one neurofibroma case, the intraspinal part of a neurofibroma was completely resected and an extraspinal part was intentionally left (case N°31). The second STR case was a neurofibroma with infiltration of the vertebral artery, so a small tumor remnant was intentionally left on the vertebral artery (case N°13). In the last case of STR, partof the schwannoma capsule was left due to strong adhesion to the visceral pleura (case N°5).

We observed no major surgical complications, no severe postoperative neurological deterioration, and no wrong level surgery. Minor complications occurred in four cases (8%), all of which were CSF fistulas. Two of these were successfully managed by lumbar drainage only, one by revision surgery and one by revision surgery combined with lumbar drainage without recurrence of CSF fistula. There were nine patients with mild postoperative hypesthesia and five patients with discrete motor weakness one grade lower than preoperatively according to the Medical Research Council (MRC) grading system. In five of the patients the postoperative hypesthesia was permanent (10%), in two of the patients the postoperative motor weakness was permanent (4%).

**Table 1:** Demographics and histology of minimally invasive resected benign nerve sheath tumors. The two patients who underwent two surgeries on different locations are labelled with * and °. BMI = body mass index

Case	Age	Sex	BMI	Pathology	Case	Age	Sex	BMI	Pathology
1	66 y	M	24.1	Schwannoma	27*	71 y	F	26.2	Neurofibroma
2	71 y	F	28.4	Schwannoma	28*	71 y	F	26.2	Schwannoma
3	71 y	F	33.3	Neurofibroma	29	54 y	F	24.2	Schwannoma
4	44 y	F	20.2	Schwannoma	30	72 y	F	23.0	Schwannoma
5	47 y	M	27.8	Schwannoma	31	51 y	F	26.6	Neurofibroma
6	46 y	F	21.5	Neurofibroma	32	57 y	F	27.4	Schwannoma
7	25 y	F	25.6	Schwannoma	33°	37 y	M	28.7	Schwannoma
8	60 y	M	33.6	Neurofibroma	34	56 y	F	27.2	Schwannoma
9	21 y	F	23.0	Neurofibroma	35	47 y	F	21.5	Neurofibroma
10	40 y	F	22.8	Schwannoma	36	68 y	M	26.8	Schwannoma
11	62 y	M	21.6	Schwannoma	37	78 y	M	33.9	Schwannoma
12	52 y	F	27.2	Schwannoma	38	38 y	M	28.3	Schwannoma
13	26 y	M	28.4	Neurofibroma	39	38 y	M	23.5	Schwannoma
14	51 y	F	29.3	Schwannoma	40	49 y	M	24.8	Schwannoma
15	55 y	F	28.4	Schwannoma	41	52 y	F	39.4	Schwannoma
16	55 y	M	24.9	Schwannoma	42°	38 y	M	28.7	Neurofibroma
17	53 y	F	29.1	Schwannoma	43	55 y	F	25.4	Schwannoma
18	49 y	F	19.4	Schwannoma	44	34 y	M	23.1	Schwannoma
19	68 y	F	39.8	Schwannoma	45	46 y	M	26.3	Schwannoma
20	37 y	M	23.4	Schwannoma	46	88 y	F	24.5	Schwannoma
21	15 y	M	21.7	Schwannoma	47	37 y	F	21.6	Schwannoma
22	40 y	M	29.5	Neurofibroma	48	54 y	M	24.2	Schwannoma
23	34 y	F	19.3	Schwannoma	49	71 y	M	26.3	Schwannoma
24	45 y	F	23.9	Schwannoma	50	63 y	F	25.0	Schwannoma
25	80 y	M	24.5	Schwannoma	51	17 y	M	23.5	Schwannoma
26	72 y	M	31.6	Schwannoma					

### Exemplary cases

**Fig. 4:**
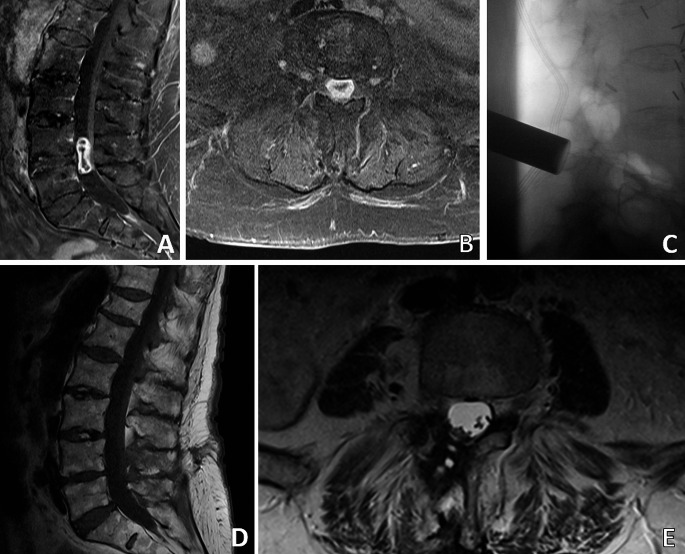
Exemplary case of an Ia classified intraspinal, intradural schwannoma (case 46). Sagittal (**A**) and axial (**B**) preoperative MRI scans show the lesion. The intraoperative X-ray shows the placement of the tubular retractor (**C**). Postoperative findings of case 46: Sagittal (Dt) and axial (Et) MRI scans show GTR

Case N°46 is a female patient, who was 88 years old at the time of the surgery. She initially presented with a two-week history of radicular pain in the left L5 region. Preoperative examination revealed a mild paresis of the left foot extensor (grade 4 according to the MRC grading system) and a mild hypaesthesia of the dorsal right foot. MRI showed an intraspinal, intradural tumor measuring 12 × 16 × 31 mm on level L4/L5, Sridhar classification Ia was found (Fig. 4). Surgery was performed via non-expandable tubular retractor (Fig. 4). Operative time was 131 min with a blood loss of 150 ml. No new deficit occurred after surgery. The mild paresis was no longer detectable. Pain completely resolved within days. The patient was discharged 6 days after surgery. Postoperative MRI showed GTR (Fig. 4). The follow-up period was 3 months. No recurrence or signs of spinal instability occurred.

**Fig. 5:**
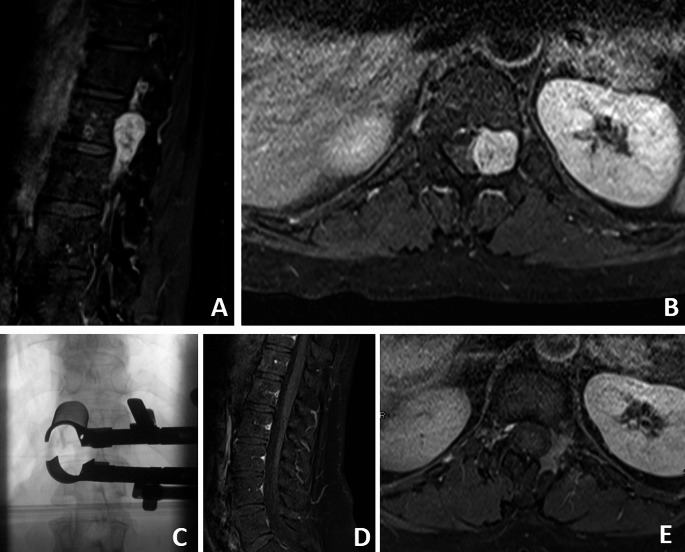
Exemplary case of an IVa classified dumbbell schwannoma (case 34). The expansion of the tumor intraforaminal is shown on the axial MRI scan (**B**). The extradural tumor mass is shown on the sagittal scan (**A**). Intraoperative X-rays of patient 34 show the retractor placement from anterior-posterior (**C**). Postoperative sagittal (**D**) and axial (**E**) MRI scans of patient 34 show a GTR of the IVa classified dumbbell schwannoma while sparing > 50% of the facet joint

Case N° 34 is a female patient aged 56 years old when she presented with radicular pain along the left L4 dermatome of 10 months’ duration. The pain intensity was VAS 4/10. Clinical examination revealed a mild paresis of the left hip flexors with the strength level of 4+/5 and a hypaesthesia in the area of the L4 dermatome on the left side. In addition, the patient showed an unsteady gait. MRI showed a dumbbell shaped tumor mass measuring 23 × 14 × 26 mm on Th 12 level, Sridhar classification IVa (Fig. 5). Surgery was performed using an expandable tubular retractor (Fig. 5). Access to the tumor was gained through a hemilaminectomy with preservation of > 50% of the facet joint. The tumor could be resected completely through the intraspinal approach since the tumor had widened the spinal nerve foramen. Operative time was 139 min, blood loss was 300 ml. Postoperatively the hypaesthesia persisted while the paresis was diminishing. No new deficit occurred. The patient was discharged 5 days after surgery, the pain level on the day of discharge was NRS 0/10. Postoperative MRI showed GTR (Fig. 5). The follow-up period was 70 months. No recurrence occurred.

**Fig. 6:**
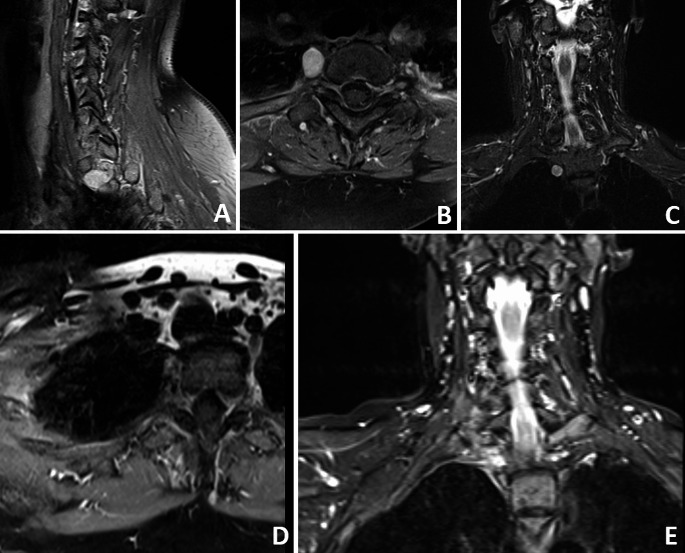
Exemplary case of a completely paraspinal schwannoma (case 29). The tumor mass is in contact with the spine as well as the apical lung as shown in the sagittal (**A**), axial (**B**), and coronar (**C**) MRI scans. GTR was achieved as the follow up MRI shows in axial (**D**) and coronar (**E**)

Case N°29 is a 54-year-old woman. A Th1 extradural paravertebral schwannoma was diagnosed 6 months after the onset of radicular pain, hypaesthesia of the right hand and a mild paresis of the interossei muscles of the right hand occurred. The tumor was 15 × 11 × 11 mm in size (Fig. 6). Surgery was performed using an expandable tubular retractor. Access to the tumor was gained via costotransversectomy. Operative time was 115 min, blood loss was 50 ml. Histology revealed a schwannoma. Postoperatively the patient showed no new deficit, the hypaesthesia and paresis was regressive. She was discharged from hospital four days later. Follow up MRI showed GTR (Fig. 6). During the follow up period of 69 months, no growth of the tumor occurred.

## Discussion

BNST are the most common spinal neoplasms and make up 30% of all spinal tumors [1]. In these 51 operated spinal and paraspinal BNST, we have shown that the minimally invasive tubular approach is safe and effective for removing these tumors to a satisfactory extent. The tailored angled positioning of the tubular retractor on the tumor (Fig. 2) could prevent a facetectomy in most of the cases. Furthermore, none of the cases required spinal fusion during a mean follow-up period of 30.8 months.

Standard open resection of BNST requires a relatively long skin incision and wide approach with associated muscle trauma and bone resection, possibly including the facet joint to obtain adequate surgical exposure. Studies comparing the minimally invasive to the open approach in spine surgery have shown that intraoperative blood loss as well as intra- and postoperative analgesia demand is lower in MIS [7, 15, 16]. Minimally invasive operated patients have a shorter length of in hospital stay and a faster return to work [5, 6, 16]. As early as 1989, Chiou et al. demonstrated in 256 cases that a smaller approach via hemilaminectomy compared to a complete laminectomy in spinal tumor resections is advantageous for the patients in terms of neurological outcome and length of hospital stay [17]. Shin et al. demonstrated an association between the reduction of iatrogenic muscle injury and postoperative pain using muscle metabolic parameters such as lactate levels and creatinine in patients who underwent micro endoscopic lumbar discectomy compared to standard microdiscectomy procedure [18]. It is well known that patients who undergo minimally invasive surgeries have less pain and less surgical trauma, making early mobilisation more feasible [16]. Therefore, complications related to bed rest are lower [19]. Than et al. stated a favourable outcome and fewer CSF leakage complications for patients using the MIS approach due to reduced potential dead space in the surgical field [20]. Fontes et al. compared the costs accruing in open and MIS removal of intradural extramedullary tumors and showed significantly lower costs with the MIS approach [9].

The literature on minimally invasive, mini open or open resections of BNST consists mainly of studies with small sample size and case reports (Table 2). Lu et al. resected three extradural foraminal tumors of the lumbar spine via mini open approach using an expandable tubular retractor to perform a hemilaminectomy and complete facetectomy [21]. They reported GTR in 2 out of 3 cases and no adverse events. However, an abnormal motion was observed in all operated segments and spinal fusion was necessary in all three patients. Vergara described one case of a lumbar both intra- and extradural schwannoma resected via a dual intra- and extraspinal mini-open approach [22]. GTR was achieved through hemilaminectomy, but since the facet joint was unimpaired, spinal fusion was not necessary. Recently, a case series of 15 giant dumbbell spinal schwannomas was published by Poblete et al. [23]. They accessed the tumors via an expandable retractor and performed partial hemilaminectomy with less than one third medial facetectomy. GTR was achieved in all cases without any adverse events or the need for bone fusion.

**Table 2:** Literature overview. The upper part shows MIS and mini open approaches, the middle part open approaches and the bottom part comparative studies. LOS = length of stay. Compl. = complications. Sp. Fusion = spinal fusion

Study	Cases	Tumor + Location	Technique	OR-Time	Blood Loss	LOS	GTR	Compl.	Sp. Fusion	Follow up
Tredway et al. [27]	6	5 intradural schwannomas, 1 intradural ependymoma	MIS tubular	247 min	56 ml	2.4 days	100%	None	0%	3–18 months
Shah et al. [11]	1	Lumbar extradural schwannoma	MIS tubular	75 min	30 ml	3 days	100%	None	0%	6 months
Lu et al. [21]	3	Lumbar extradural foraminal	Mini open	200 min	250 ml	3.3 days	66%	None	100%	14–24 mon.
Haji et al. [25]	20	Mixed spinal pathologies	MIS tubular	210 min	428 ml	3 days	68%	9%	0%	6 weeks
Mannion et al. [26]	13	Intradural spinal tumors	MIS tubular	150 min	155 ml	3.1 days	92%	15%	0%	n.a.
Weil et al. [27]	1	Lumbar extradural schwannoma	MIS tubular	-	-	2 days	100%	None	0%	6 months
Nozokou [24]	13	Thoracic and lumbar extradural schwannomas	MIS tubular	189 min	219 ml	2.8 days	92%	None	0%	21 months
Goncalves et al. [4]	1	Far lateral extradural lumbar schwannoma	MIS tubular	170 min	300 ml	2 days	100%	None	0%	18 months
Tan et al. [29]	23	Diverse intradural pathologies	MIS tubular	161 min	107 ml	3.3 days	100%	None	0%	21 months
Vergara [22]	1	Lumbar mixed intra/extradural schwannoma	Mini open	-	-	-	100%	None	0%	14 months
Wang et al. [33]	46	Lumbar dumbbell mixed pathologies	MIS tubular	110 min	85 ml	6 days	100%	21.7%	0%	27.3 months
Poblete et al. [23]	15	Giant dumbbell schwannomas	Mini open	-	-	2.6 days	100%	None	0%	10.4 mon.
Argiti et al. 2024 (this series)	51	BNST of all locations	MIS tubular	167 min	138 ml	5.3 days	86% GTR	8%	0%	30.8 months
Chiou et al. [17]	256	Diverse spinal tumors	Open	-	-	8.5 days	61%	12%	-	8.6 months
Deng et al. [30]	52	Spinal schwannoma	Open I/II	I 144 min II 168 min	I 300 ml II 561 ml	I 8.4 days II 10.4 days	-	-	100%	6–36 months
Chang et al. [32]	7	Dumbbell schwannoma	Open	-	-	-	71%	14%	0%	23 months
Parlak et al. [31]	50	Spinal schwannoma	Open	-	-	-	76%	10%	2%	30 months
Lee et al. [15]	49	Spinal schwannoma	I MIS tubular II Open	I 127 min II 174 min	I 207 ml II 427 ml	I 3.8 days II 6.8 days	I 100% II 87.5%	I 0% II 6%	I 0% II 19%	I 38 months II 51 mon.
Wong et al. [33]	45	Intradural spinal Tumors	I MIS tubular II open	I 256 min II 241 min	I 134 ml II 559 ml	I 3.9 days II 6.1 days	I 92.6% II 94.4%	I 11% II 22%	I 0% II 11%	n.a.

The largest case series we found for tubular retractor-based MIS of BNST was published by Nzokou [24]. 13 cases of thoracic and lumbar extradural schwannomas were operated via a tubular retractor. GTR was achieved in 12 of the patients, there was no need for spinal fusion and no adverse events were reported. Haji et al. performed MIS resection of 20 intra- and extradural schwannomas, meningiomas, ependymomas, and teratomas via hemilaminectomy [25]. In total, 68% of all cases had GTR. Only extradural schwannomas had 100% GTR. In a case series published by Mannion et al., 13 intradural spinal tumors were resected via expandable or non-expandable tubular retractor [26]. GTR was achieved in 12 of the patients. Tredway et al. described 6 cases of intradural schwannomas and ependymomas resected via tubular retractor and one or two level hemilaminectomy [27]. GTR was achieved in all cases and no adverse events were reported. Shah, Weil and Goncalves reported one case each: Goncalves et al. resected a far lateral lumbar schwannoma via expandable tubular retractor without complications [4]. Shah et al. and Weil et al. both reported each one case of a lumbar extradural schwannoma that was resected via tubular retractor [11, 28]. In both cases GTR was achieved and no complications were reported. Tan et al. reported a series of resections of 23 intradural pathologies, including neoplastic, congenital, vascular, and degenerative lesions [29]. GTR was achieved in all patients and no complications were reported.

Larger patient cohorts have been reported for open resection of BNST. In 1989, Chiou et al. published a retrospective analysis of 256 patients who underwent open surgery for spinal tumors, including metastasis, meningioma, neurinoma, ependymoma, and astrocytoma [17]. 61% of the tumors were completely removed. Complications were reported in 12% of the cases. Another retrospective analysis was performed by Deng et al. who analysed 52 patients with spinal schwannoma [30] resected in open surgery via hemilaminectomy or laminectomy. All patients underwent internal fixation with pedicle screws. The extent of resection and complications were not reported. Parlak et al. analysed 50 cases of spinal schwannoma resections via laminotomy (72%) or hemilaminectomy (24%) [31]. GTR was achieved in 76%, with complications in 10% of the cases. Chang et al. performed open surgery on 7 patients with dumbbell spinal schwannomas via unilateral or bilateral laminectomy and achieved GTR in 71% [32].

There are no randomized controlled trials comparing open with MIS resection of BNST. However, there are few nonrandomized studies comparing open surgery to a MIS approach. Lee et al. retrospectively analysed 49 patients with spinal schwannoma who underwent surgery either with a MIS or traditional open approach [15]. Two different MIS techniques were used: Hemilaminectomy through a non-expandable tubular retractor or unilateral muscle retraction. More precisely, the second `MIS` approach involved open access to the bone structures via unilateral subperiosteal muscle dissection from the spinous process to lamina with subsequent hemilaminectomy. These two described MIS approaches show the range of different techniques that are subsumed under the term MIS. Notably, in this study all surgeries were performed by the same surgeon. The comparison was made between MIS and open, without considering the different MIS techniques. Blood loss, operative time and length of stay were higher in patients who underwent open surgery, while the extent of resection and complication rate were comparable. Patients who underwent open surgery were more likely to require spinal fusion.

Wong et al. published a case series of 45 patients with spinal tumors, including 62% nerve sheath tumors, 22% meningiomas, and 7% congenital lesions [7]. Surgery was performed either via open approach or via expandable tubular retractor. The results demonstrate a significantly lower blood loss, operative time, and length of stay in patients undergoing MIS. Complication rates were also lower. In the MIS cases no blood transfusions were necessary, while there were transfusions in 11% of the cases in the open surgery group. Spinal fusion was necessary in 11% of the open surgery group.

Recently, Wang et al. published a series of 46 patients with lumbar dumbbell shaped tumors of mixed benign and malignant pathologies [33]. All tumors were resected via tubular retractor. The authors report total resection in 100% of the cases.

Ultimately, when comparing operative characteristics and outcomes of the studies above, MIS resection of spinal BNST is feasible and safe. While operative time, blood loss and length of stay is significantly lower in MIS than in open surgery, complications, and STR rates remain comparable.

The limitations of most trials are small patient numbers and selection bias. Most studies only focus on a specific type of BNST or on one location only. Compared to those studies, our patient collective is the most heterogenous, including both schwannomas as well as neurofibromas of all sizes, shapes, and spinal and paraspinal locations throughout the spine.

To our knowledge this is the largest report to date of patients undergoing MIS to describe a safe and conclusive technique for removal of spinal and paraspinal BNST. Limitations of this study include its retrospective nature and the lack of an open approach control group with which to compare surgical outcomes such as GTR rates and postoperative CSF-related complications. A preoperative selection bias must also be considered.

A disadvantage of MIS is that not only the retractor systems, but also the bayonet style instruments, and microscopy are necessary (see “surgical technique”). The smaller the approach, the more limited the view. To our experience, the learning curve for MIS via retractor systems is steep. Nevertheless, the surgeon needs to be familiar with this technique. We recommend the application of this technique in less complex extradural procedures, e.g. lumbar disc herniation, before using it on intradural tumor resection. Especially dural closure can be challenging through narrow and long retractor systems. On the other hand, Kogias et al. found that in cases of incidental durotomy postoperative CSF-fistulas were less likely in MIS compared to open surgery, probably due to less dead space through the narrow approach [34].

The fact that we were able to perform MIS resection of BNST safely and effectively, together with the knowledge that minimally invasive spine surgery has advantages over standard open approaches in terms of operating time, blood loss, complications, early mobilization, analgetic use and length of in hospital stay, suggests that MIS may also be beneficial in selected patients with BNST.

Prospective studies are undoubtedly desirable and represent the logical next step in our research.

Finally, it should be noted that there is no general definition of an MIS approach to the spine. Many of the studies mentioned above showed results of different surgical techniques, all of which claim to be minimally invasive. Comparing the different strategies reveals the multiple nuances of MIS. In order to determine the superiority of one approach, clearer definitions of MIS approaches would be desirable.

## Conclusions

Spinal schwannoma and neurofibroma can be removed effectively and safely through a minimally invasive tubular approach, with satisfactory extent of tumor resection and no increased risk for neurological deterioration.

## Electronic supplementary material

Below is the link to the electronic supplementary material.

Supplementary Material 1: Surgery data.

Supplementary Material 2: Tumor characteristics.

Supplementary Material 3

## Data Availability

Data is provided within the manuscript or supplementary information files. Also raw data can be obtained via email to the corresponding author.
