# Supplementary material for: Minimally invasive tubular removal of spinal schwannoma and neurofibroma - a case series of 49 patients and review of the literature
Source: Neurosurg Rev. 2024 Aug 10;47(1):418. doi: 10.1007/s10143-024-02656-x (PMC11315786; doi:10.1007/s10143-024-02656-x)
Supplement: Supplementary file 1 — Supplementary Material 1: Surgery data. [file 10143_2024_2656_MOESM1_ESM.docx]

| **Case** | **Level** | **Location 1** | **Location 2** | **Sridhar Class.** | **Size [mm]** | **Volume [cm³]** | **Pathology** |
| --- | --- | --- | --- | --- | --- | --- | --- |
| 1 | L 4 | Dumbbell | Extradural | IV a | 21x16x12 | 2.11 | Schwannoma |
| 2 | Th 12 | Intraspinal | Intradural | I a | 28x17x16 | 3.99 | Schwannoma |
| 3 | L 4 | Intraforaminal | Extradural | III | Missing data | / | Neurofibroma |
| 4 | L 2 | Intraforaminal | Extradural | III | Missing data | / | Schwannoma |
| 5 | Th 1 | Extraspinal | Extradural | IV b | 34x30x38 | 20.29 | Schwannoma |
| 6 | Th 3 | Extraspinal | Extradural | IV a | 20x19x15 | 2.98 | Neurofibroma |
| 7 | Th 12 | Dumbbell | Extradural | IV b | 26x24x20 | 6.54 | Schwannoma |
| 8 | L 5 | Paraspinal | Extradural | Paraspinal | 18x15x15 | 2.12 | Neurofibroma |
| 9 | Th 1 | Dumbbell | Extradural | IV b | 47x38x33 | 30.86 | Neurofibroma |
| 10 | L 5 | Dumbbell | Extradural | IV b | 26x21x20 | 5.72 | Schwannoma |
| 11 | Th 12 | Intraspinal | Intradural | I a | 11x11x10 | 0.63 | Schwannoma |
| 12 | L 3 | Dumbbell | Extradural | III | 27x25x9 | 3.18 | Schwannoma |
| 13 | C 3 | Dumbbell | Mixed | IV a | 26x11x8 | 1.20 | Neurofibroma |
| 14 | C 5 | Dumbbell | Mixed | IV b | 24x13x14 | 2.29 | Schwannoma |
| 15 | L 3 | Intraspinal | Intradural | I a | 12x12x17 | 1.28 | Schwannoma |
| 16 | Th 11 | Intraspinal | Intradural | I a | 7x5x6 | 0.11 | Schwannoma |
| 17 | Th 10 | Dumbbell | Mixed | IV a | 8x11x24 | 1.11 | Schwannoma |
| 18 | S 1 | Dumbbell | Mixed | IV a | 24x24x24 | 7.24 | Schwannoma |
| 19 | L 1 | Intraspinal | Intradural | I a | 10x8x9 | 0.47 | Schwannoma |
| 20 | L 4 | Intraspinal | Intradural | IV a | 21x6x11 | 0.73 | Schwannoma |
| 21 | C 7 | Dumbbell | Mixed | IV b | 46x25x18 | 10.84 | Schwannoma |
| 22 | L 3 | Intraforaminal | Extradural | III | 21x14x23 | 3.54 | Neurofibroma |
| 23 | S 1 | Intraspinal | Intradural | I a | 16x12x12 | 1.21 | Schwannoma |
| 24 | L 3 | Dumbbell | Intradural | IV a | 21x23x11 | 2.78 | Schwannoma |
| 25 | C 7 | Intraspinal | Intradural | II a | 17x8x13 | 0.93 | Schwannoma |
| 26 | Th 12 | Intraspinal | Intradural | I a | 22x10x9 | 1.03 | Schwannoma |
| 27 | Th 9 | Paraspinal | Extradural | Paraspinal | 22x22x20 | 5.07 | Neurofibroma |
| 28 | L 3 | Intraspinal | Intradural | I a | 8x6x7 | 0.18 | Schwannoma |
| 29 | Th 1 | Paraspinal | Extradural | Paraspinal | 15x11x11 | 0.95 | Schwannoma |
| 30 | L 3 | Intraforaminal | Extradural | IV a | 12x12x19 | 1.43 | Schwannoma |
| 31 | L 5 | Dumbbell | Intradural | IV b | 32x14x15 | 13.19 | Neurofibroma |
| 32 | L 1 / Cauda | Intraspinal | Intradural | I a | 24x17x17 | 3.61 | Schwannoma |
| 33 | L 5 | Intraspinal | Intradural | I a | 6x6x8 | 0.15 | Schwannoma |
| 34 | Th 12 | Dumbbell | Intradural | IV a | 23x14x26 | 4.76 | Schwannoma |
| 35 | L 4 | Dumbbell | Extradural | V | 55x40x30 | 34.56 | Neurofibroma |
| 36 | L 2 | Dumbbell | Extradural | IV a | 20x15x27 | 4.24 | Schwannoma |
| 37 | C 3 | Intraspinal | Intradural | III | 39x18x11 | 4.04 | Schwannoma |
| 38 | L 5 | Intraspinal | Intradural | I a | 14x12x25 | 2.20 | Schwannoma |
| 39 | L 4 | Dumbbell | Extradural | IV a | 20x12x14 | 1.76 | Schwannoma |
| 40 | C 8 | Intraspinal | Intradural | I a | 14x8x14 | 0.82 | Schwannoma |
| 41 | Th 12 | Intraforaminal | Extradural | III | 23x11x21 | 2.78 | Schwannoma |
| 42 | L 1 | Intraspinal | Intradural | I a | 10x10x8 | 0.42 | Neurofibroma |
| 43 | L 5 | Intraspinal | Intradural | III | 30x8x12 | 1.15 | Schwannoma |
| 44 | C 4 | Dumbbell | Intradural | IV a | 33x12x12 | 2.49 | Schwannoma |
| 45 | L 3 | Intraspinal | Intradural | I a | 16x13x12 | 1.31 | Schwannoma |
| 46 | L 4 | Intraspinal | Intradural | I a | 12x16x31 | 3.12 | Schwannoma |
| 47 | L 5 | Intraspinal | Intradural | I a | 13x12x11 | 0.90 | Schwannoma |
| 48 | C 6 | Intraspinal | Intradural | III | 10x21x8 | 0.88 | Schwannoma |
| 49 | C 4 | Intraspinal | Intradural | I a | 15x14x8 | 0.88 | Schwannoma |
| 50 | L 1 | Intraspinal | Intradural | I a | 8x8x10 | 0.34 | Schwannoma |
| 51 | S 1 | Intraspinal | Intradural | IV b | 19x34x19 | 6.43 | Schwannoma |

Supplement Table B: Tumor characteristics including Sridhar classification.
