# Supplementary material for: Minimally invasive tubular removal of spinal schwannoma and neurofibroma - a case series of 49 patients and review of the literature
Source: Neurosurg Rev. 2024 Aug 10;47(1):418. doi: 10.1007/s10143-024-02656-x (PMC11315786; doi:10.1007/s10143-024-02656-x)
Supplement: Supplementary file 2 — Supplementary Material 2: Tumor characteristics. [file 10143_2024_2656_MOESM2_ESM.docx]

| **Case** | **Cut length** | **Retractor** | **Surgical technique** | **Neuro monitoring** | **Surg. Time [min]** | **Blood loss [ml]** | **Spinal fusion** | **Dura closure** | **Resection** |
| --- | --- | --- | --- | --- | --- | --- | --- | --- | --- |
| 1 | 2.0 cm | Expandable | Partial facetectomy | No | 135 | 200 | No | Suture | GTR |
| 2 | 3.5 cm | Non-expandable | Partial hemilaminectomy | No | 133 | 200 | No | Suture + fibrin glue + Gelita | GTR |
| 3 | n.a. | Expandable | Partial hemilaminectomy | No | 113 | 50 | No | Suture | GTR |
| 4 | 2.0 cm | Expandable | Facetectomy | No | 83 | 200 | No | Suture | GTR |
| 5 | n.a. | Non-expandable | Costotransversectomy | No | 255 | 50 | No | Suture | STR |
| 6 | 2.5 cm | Expandable | Facetectomy | No | 123 | 50 | No | Suture | GTR |
| 7 | 3.5 cm | Non-expandable | Extraforaminal approach | No | 136 | 50 | No | / | GTR |
| 8 | n.a. | Expandable | Extraspinal approach | Yes | 97 | 50 | No | / | GTR |
| 9 | 4.5 cm | Non-expandable | Costotransversectomy | Yes | 217 | 100 | No | Suture | GTR |
| 10 | 2.5 cm | Expandable | Extraforaminal approach | Yes | 116 | 50 | No | / | GTR |
| 11 | 3.0 cm | Non-expandable | Hemilaminectomy | No | 129 | 50 | No | Suture + Tachosil | GTR |
| 12 | 4.0 cm | Non-expandable | Partial hemilaminectomy | Yes | 136 | 50 | No | Suture | GTR |
| 13 | 2.5 cm | Expandable | Partial hemilaminectomy | No | 168 | 300 | No | Suture + Duraform + Tachosil | STR |
| 14 | n.a. | Non-expandable | Partial hemilaminectomy | Yes | 170 | 50 | No | Suture + Tachosil | GTR |
| 15 | n.a. | Non-expandable | Hemilaminectomy | Yes | 207 | 150 | No | Suture + Tachosil + fibrin glue | GTR |
| 16 | 3.0 cm | Non-expandable | Partial hemilaminectomy | No | 73 | 50 | No | Suture + Tachosil + Gelita | GTR |
| 17 | 3.0 cm | Non-expandable | Partial hemilaminectomy | No | 129 | 50 | No | Suture + Tachosil + Gelita | GTR |
| 18 | 3.0 cm | Non-expandable | Partial hemilaminectomy | Yes | 167 | 500 | No | Suture + Gelita | GTR |
| 19 | 4.0 cm | Non-expandable | Hemilaminectomy | No | 295 | 350 | No | Suture + Tachosil | GTR |
| 20 | n.a. | Expandable | Partial hemilaminectomy | Yes | 126 | 50 | No | Suture + Tachosil + Gelita | GTR |
| 21 | 3.0 cm | Non-expandable | Hemilaminectomy | Yes | 264 | 50 | No | Duragen + fibrin glue + autologous fat | GTR |
| 22 | n.a. | Expandable | Extraspinal approach | Yes | 171 | 50 | No | / | GTR |
| 23 | 2.5 cm | Expandable | Partial hemilaminectomy | Yes | 117 | 50 | No | Suture + fibrin glue + Gelita | GTR |
| 24 | n.a. | Expandable | Partial hemilaminectomy | Yes | 103 | 50 | No | Suture + + fibrin glue + autologous fat | GTR |
| 25 | 4.0 cm | Expandable | Hemilaminectomy | Yes | 192 | 100 | No | Suture | GTR |
| 26 | 4.0 cm | Non-expandable | Hemilaminectomy | Yes | 197 | 50 | No | Suture + Tachosil + Gelita | GTR |
| 27 | 4 cm | Expandable | Facetectomy | No | 91 | 100 | No | Suture + Gelita | GTR |
| 28 | 3 cm | Expandable | Partial hemilaminectomy | Yes | 126 | 100 | No | Suture + Duraseal + Gelita | GTR |
| 29 | n.a. | Non-expandable | Costotransversectomy | No | 115 | 50 | No | / | GTR |
| 30 | 1.8 cm | Non-expandable | Extraforaminal approach | Yes | 70 | 50 | No | Suture + Gelita | GTR |
| 31 | 3.5 cm | Expandable | Hemilaminectomy/Facetectomy | Yes | 364 | 800 | No | Suture + Tachosil + Gelita | STR |
| 32 | 3 cm | Expandable | Hemilaminectomy | Yes | 139 | 50 | No | Suture | GTR |
| 33 | 3.5 cm | Expandable | Hemilaminectomy | Yes | 159 | 300 | No | Suture + fibrin glue + Gelita | GTR |
| 34 | n.a. | Expandable | Hemilaminectomy | Yes | 139 | 300 | No | Suture + Tachosil | GTR |
| 35 | 4 cm | Expandable | Extraspinal approach | Yes | 266 | 170 | No | Suture + Gelita | GTR |
| 36 | 4 cm | Expandable | Extraforaminal approach | Yes | 152 | 50 | No | Suture + Gelita | GTR |
| 37 | 4 cm | Expandable | Hemilaminectomy | Yes | 230 | 50 | No | Suture + fibrin glue + Gelita | GTR |
| 38 | 2.5 cm | Expandable | Partial hemilaminectomy | Yes | 113 | 50 | No | Suture + fibrin glue + Gelita | GTR |
| 39 | 3 cm | Non-expandable | Extraforaminal approach | No | 146 | 50 | No | Suture + Gelita | GTR |
| 40 | n.a. | Expandable | Hemilaminectomy | Yes | 262 | 100 | No | Suture + Tachosil + Tabotamp | GTR |
| 41 | 4 cm | Expandable | Partial hemilaminectomy, facetectomy | No | 79 | 50 | No | / | GTR |
| 42 | 3 cm | Expandable | Hemilaminectomy | Yes | 136 | 100 | No | Suture + Tachosil | GTR |
| 43 | 3.5 cm | Expandable | Hemilaminectomy | Yes | 177 | 60 | No | Suture + Tisseal | GTR |
| 44 | n.a. | Expandable | Hemilaminectomy | Yes | 330 | 400 | No | Suture + Gelita + Tissuecol + fibrin glue | GTR |
| 45 | n.a. | Expandable | Partial hemilaminectomy | Yes | 198 | 300 | No | Suture + Tachosil | GTR |
| 46 | 2.5 cm | Non-expandable | Hemilaminectomy | Yes | 131 | 150 | No | Suture + Tachosil + Gelita | GTR |
| 47 | n.a. | Expandable | Partial hemilaminectomy | Yes | 188 | 200 | No | Suture + fibrin glue + Gelita | GTR |
| 48 | 3 cm | Expandable | Partial hemilaminectomy | Yes | 239 | 200 | No | Suture + Duragen + Gelita | GTR |
| 49 | 1.5 cm | Non-expandable | Hemilaminectomy | Yes | 125 | 50 | No | Suture + Tachosil | GTR |
| 50 | 2 cm | Non-expandable | Hemilaminectomy | Yes | 173 | 50 | No | Suture | GTR |
| 51 | n.a. | Expandable | Partial hemilaminectomy | Yes | 283 | 300 | No | Suture + fibrin glue + Gelita + Tachosil | GTR |

Supplement Table A: Surgery data
